# Toward a realistic in silico abdominal phantom for QSM

**DOI:** 10.1002/mrm.29597

**Published:** 2023-01-25

**Authors:** Javier Silva, Carlos Milovic, Mathias Lambert, Cristian Montalba, Cristóbal Arrieta, Pablo Irarrazaval, Sergio Uribe, Cristian Tejos

**Affiliations:** ^1^ Department of Electrical Engineering Pontificia Universidad Católica de Chile Santiago Chile; ^2^ Biomedical Imaging Center Pontificia Universidad Católica de Chile Santiago Chile; ^3^ Millennium Institute for Intelligent Healthcare Engineering (iHEALTH) Santiago Chile; ^4^ School of Electrical Engineering Pontificia Universidad Católica de Valparaíso Valparaíso Chile; ^5^ Department of Radiology, School of Medicine Pontificia Universidad Católica de Chile Santiago Chile; ^6^ Institute for Biological and Medical Engineering, Pontificia Universidad Católica de Chile Santiago Chile

**Keywords:** abdomen, digital phantom, liver QSM, MRI simulation, quantitative susceptibility mapping, water‐fat separation

## Abstract

**Purpose:**

QSM outside the brain has recently gained interest, particularly in the abdominal region. However, the absence of reliable ground truths makes difficult to assess reconstruction algorithms, whose quality is already compromised by additional signal contributions from fat, gases, and different kinds of motion. This work presents a realistic in silico phantom for the development, evaluation and comparison of abdominal QSM reconstruction algorithms.

**Methods:**

Synthetic susceptibility and $R_{2}^{*}$ maps were generated by segmenting and postprocessing the abdominal 3T MRI data from a healthy volunteer. Susceptibility and $R_{2}^{*}$ values in different tissues/organs were assigned according to literature and experimental values and were also provided with realistic textures. The signal was simulated using as input the synthetic QSM and $R_{2}^{*}$ maps and fat contributions. Three susceptibility scenarios and two acquisition protocols were simulated to compare different reconstruction algorithms.

**Results:**

QSM reconstructions show that the phantom allows to identify the main strengths and limitations of the acquisition approaches and reconstruction algorithms, such as in‐phase acquisitions, water‐fat separation methods, and QSM dipole inversion algorithms.

**Conclusion:**

The phantom showed its potential as a ground truth to evaluate and compare reconstruction pipelines and algorithms. The publicly available source code, designed in a modular framework, allows users to easily modify the susceptibility, $R_{2}^{*}$ and TEs, and thus creates different abdominal scenarios.

## Abstract 2

Click here for author‐reader discussions

## 1 INTRODUCTION

QSM is a technique that computes the magnetic susceptibility of tissues from small magnetic field variations encoded in the phase signal of a MR image.^1^, ^2^, ^3^, ^4^ Over the past decade, QSM has proven to be a promising tool for various brain applications, including the assessment of iron deposits in deep gray matter,^2^, ^5^ demyelination in white matter,^2^ differentiation of blood products and calcifications,^4^, ^6^ estimation of vessel oxygenation and geometry,^7^, ^8^ and its use as a potential biomarker of several neurodegenerative diseases.^2^, ^4^, ^5^, ^9^, ^10^, ^11^ Recently, QSM has gained interest for applications outside the brain, for example, as a biomarker for hepatic iron overload^12^, ^13^ and fibrosis,^14^, ^15^ and chronic kidney disease.^16^

Unlike in the brain, the application of QSM in the abdomen involves a series of additional challenges and restrictions^12^, ^14^, ^17^: reduced acquisition times and resolution, undesired signal contributions from fat and gases, rapid signal decay in tissues with high iron concentrations, and different kinds of motion. These considerations result in restrictive acquisition protocols and additional preprocessing steps, which might degrade the quality of the QSM reconstructions in the abdominal area. For the case of fat contributions, abdominal QSM studies have proposed different approaches to address this problem, including in‐phase^17^, ^18^, ^19^, ^20^ or fat‐suppressed acquisitions,^21^ water and fat separation approaches, such as Iterative Graph Cuts (IGC),^22^ Simultaneous Phase Unwrapping and Removal of Chemical Shift (SPURS),^23^ or Simultaneous Multiple Resonance Frequency imaging (SMURF).^24^

Regardless of the chosen approach, abdominal QSM reconstructions tend to be over‐regularized to minimize artifacts, resulting in maps with low details that only provide significant information for severe conditions or diseases.^12^, ^14^, ^16^, ^25^ Furthermore, the absence of a reliable ground‐truth hinders the development of a more precise and effective dipole inversion approach for abdominal applications that could effectively manage such specific limitations. On the one hand, in vivo abdominal ground‐truth data could not be developed using methods like COSMOS^26^ or STI,^27^ given the unfeasibility to acquire multiple orientation images in the abdomen. Additionally, questions have been raised about the validity of using multi‐orientation reconstructions as ground‐truth due to the presence of unaccounted anisotropic contributions.^28^ On the other hand, experimental phantoms, made of bottles and vials with known water, fat, and susceptibility distributions,^29^, ^30^ provide over‐simplistic scenarios in terms of geometries and textures, which widely differ from the real anatomy.

Another way to evaluate QSM reconstruction algorithms has been done using numerical phantoms based on tissue properties derived from in vivo acquisitions.^31^, ^32^, ^33^, ^34^, ^35^ This alternative has shown to be more reliable than using real‐world data since it avoids the discrepancies generated by noise and errors propagated from earlier pre‐processing steps (i.e., coil combination inaccuracies, leftover phase wraps, residual background fields, etc.).^28^, ^35^ However, current abdominal phantoms consist in anatomical structures with piecewise susceptibility values.^19^, ^36^ This approach leads to inaccurate over‐regularized results, which are not reliable for the assessment of QSM reconstructions.

In this work, we present a realistic in silico phantom of the abdomen, which could be used to develop, evaluate, and compare the performance of abdominal QSM reconstruction algorithms. The phantom is provided with three tissue properties: proton density fat fraction (PDFF), susceptibility map, and effective transverse relaxation rate ($R_{2}^{*}$) map, where the last two can be easily adjusted to compare the algorithms under different scenarios. These properties allow simulating various disease stages and water and fat signal acquisitions at different TEs, enabling the comparison of abdominal QSM reconstruction schemes, as well as water‐fat separation approaches.

## 2 METHODS

**FIGURE 1:**
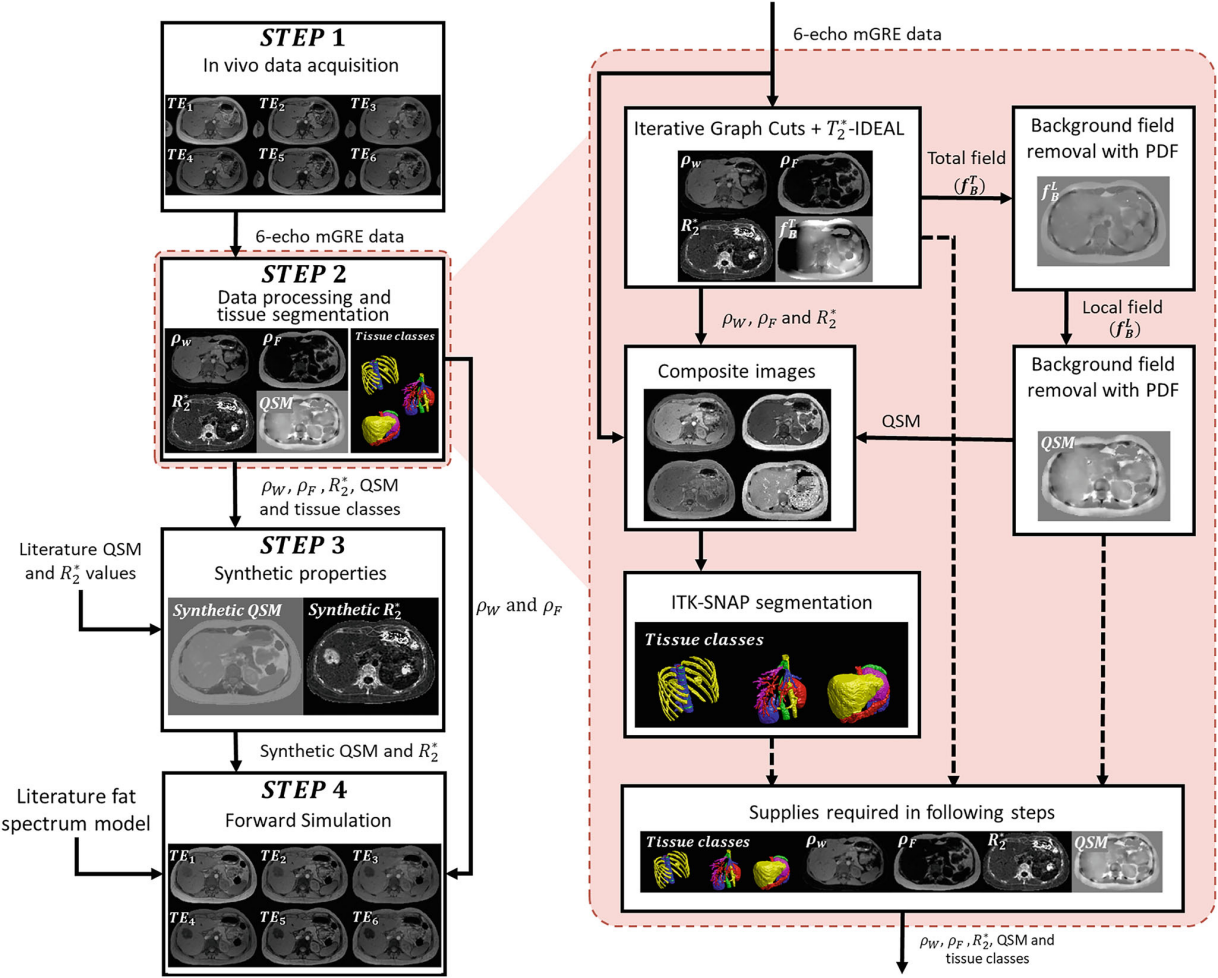
(Left) Pipeline employed in the phantom generation process. (Right) A magnified view shows the detailed workflow of the data processing and tissue segmentation step

To build our in silico abdominal phantom, we followed a similar approach as that used in the QSM Reconstruction Challenge 2.0 (RC2).^35^ Particularly, we obtained the anatomy from an MRI scan of a healthy volunteer using a six‐echo gradient recalled echo (GRE) acquisition. We employed water‐fat separation,^22^ background field removal,^37^ and dipole inversion^34^ algorithms to obtain reference values of different tissue properties from the MR images: water and fat images ($\rho_{\text{water}}$ and $\rho_{\mathrm{fat}}$), $R_{2}^{*}$ map, and QSM. Tissue property images were combined with magnitude images of the acquired data, to ease the segmentation of the different tissues of interest. Using experimental properties and reference values from the literature, we generated synthetic susceptibility and $R_{2}^{*}$ maps. Finally, water and fat images and the synthetic maps were used as inputs in a signal model that incorporates the fat contributions. The pipeline of the phantom generation is shown in Figure 1, and a detailed description of each step is presented in the following subsections.

### 2.1 In vivo data acquisition

We scanned a healthy volunteer (24‐y‐old female) in a 3T Philips Ingenia MRI scanner using an 18‐s single breath‐hold multi‐echo 3D GRE sequence. The acquisition parameters were: number of echoes = 6, TE_1_ = 1.8 ms, Δ TE = 1.56 ms, TR = 10.76 ms, flip angle = 10°, FOV = 380 × 380 × 180 mm^3^, matrix size = 192 × 192 × 90, voxel size = 1.98 × 1.98 × 2 mm^3^, bandwidth = 2222 Hz/pixel, and SENSE reduction factor = 2. The acquisition was performed after an informed consent was signed by the volunteer and under the approval of the Institutional Ethics Committee.

### 2.2 Data processing and tissue segmentation

The main purpose of this step is to obtain the inputs employed in the generation of the synthetic properties and in the forward simulation. The experimental MR properties ($\rho_{\text{water}}$, $\rho_{\mathrm{fat}}$, $R_{2}^{*}$, and QSM) and the tissue class labels were obtained following a series of interconnected processes (Figure 1, right hand side). Phase unwrapping and chemical shift removal of the acquired GRE MR images were performed using the IGC approach from the ISMRM fat‐water toolbox.^38^ The $R_{2}^{*}$, $\rho_{\text{water}}$, $\rho_{\mathrm{fat}}$, and a refined $B_{0}$ fieldmap were obtained using $T_{2}^{*}$‐IDEAL with a six‐peak fat model.^38^, ^39^

Additionally, we decided to compute a QSM from experimental data to (i) use it as an auxiliary segmentation reference for hepatic vessels, (ii) to provide susceptibility values for non‐reported tissues, and (iii) for visual comparison with our synthetic phantom. First, we generated a binary mask using the combined $\rho_{\text{water}}$, $\rho_{\mathrm{fat}}$, and echo magnitude images, excluding the arms and the internal air regions from lungs and gastrointestinal tract. Then, we applied the projection onto dipole fields (PDF) algorithm^37^ to remove the background contributions from the $B_{0}$ fieldmap derived from the water‐fat separation process. No further erosion processes were made. We performed the dipole inversion using FAst Nonlinear Susceptibility Inversion (FANSI).^34^

Composite images were generated using the magnitude images of the echoes, $\rho_{\text{water}}$ and $\rho_{\mathrm{fat}}$, QSM, and $R_{2}^{*}$ maps. These images were employed to perform the tissue segmentations with the ITK‐Snap (version 3.8) active contours function.^40^ We automatically segmented 23 structures of the abdominal region and we manually refined them. The segmented structures were the aorta, inferior vena cava, right kidney, left kidney, liver, spleen, gallbladder, esophagus, stomach, pancreas, small intestine, large intestine, hepatic veins, portal vein, ribs, vertebrae, intervertebral disks, spinal cord, subcutaneous fat, visceral fat, heart, muscle, and air regions inside the gastrointestinal tract and lungs.

### 2.3 Synthetic properties

We used the segmentations and properties obtained in the previous stage to generate two synthetic maps: susceptibility and $R_{2}^{*}$. In both cases, the map values were assigned within a similar range as that provided by the reference images (experimental QSM and $R_{2}^{*}$ maps) and the literature (healthy or pathologic cases), while increasing textures and structural details.

#### 2.3.1 Synthetic susceptibility map

A series of spherical ROIs, with radii from three to six pixels, were defined to compute the mean experimental susceptibility of each tissue class (Experimental QSM in Table 1). These ROIs were defined over regions with smooth and stable values, away from streaking artifacts or residual background fields. Air regions and large intestine were masked out in this process. The heart was also excluded to avoid cardiac motion artifacts. Muscle tissue was considered as the zero‐reference under the assumption that it does not accumulate iron,^15^, ^16^, ^41^ and the susceptibility of the esophagus was set to 0, assuming that it is only made of muscular tissue.

**TABLE 1:** Reference values and model parameters used to generate the synthetic susceptibility phantoms HS and PL

	Reference values		Synthetic susceptibility model				
	Reported	Experimental	HS	PL			
Tissue	QSM (ppm)	QSM (ppm)	$\bar{\chi_{t}}$(ppm)	$\bar{\chi_{t}}$(ppm)	$a_{t}$(ppm)	$b_{t}$(ppm)	$c_{t}$(ppm)
Aorta	−0.085^41^	0.044 ± 0.056	−0.050	0.088	0.151	−0.412	0.292
Inferior vena cava	0.054 — 0.445^4^, ^63^	0.424 ± 0.035	0.424	0.295	−0.271	0.231	1.176
Right kidney	−0.03 — 0.11^16^	0.252 ± 0.144	0.180	0.255	0.091	−1.437	0.111
Left kidney	−0.25 — 0.13^16^	0.189 ± 0.061	0.150	0.257	0.472	−0.874	0.271
Liver	0.17 — 0.23^15^, ^16^, ^41^	0.123 ± 0.121	0.150	0.182	−0.111	0.573	0.854
Spleen	NR	0.131 ± 0.129	0.130	0.181	0.091	0.030	0.653
Gallbladder	NR	0.179 ± 0.052	0.180	0.204	−0.030	−0.030	1.156
Esophagus	NR	0.164 ± 0.062	0	0.125	0.171	−0.653	0.271
Stomach	NR	0.170 ± 0.117	0.170	0.126	−0.050	−0.050	0.452
Pancreas	NR	0.074 ± 0.179	0.070	0.096	0.352	−1.015	−0.010
Small intestine	NR	0.172 ± 0.126	0.180	0.159	0.111	−0.513	0.312
Large intestine	4.84^64^	—	4.84	4.84	0	0	0
Hepatic veins	0.054 — 0.445^4^, ^63^	0.367 ± 0.068	0.367	0.309	−0.472	0.332	−0.794
Porta vein	0.054 — 0.445^4^, ^63^	0.343 ± 0.040	0.343	0.266	−0.131	0.814	1.096
Ribs	−2.4 — −1.4^35^, ^65^	−0.901 ± 0.248	−1.200	−0.109	0.030	0.412	0.975
Vertebrae	−0.037 — 0.032^66^	−0.372 ± 0.250	−0.050	−0.004	−0.111	0.251	1.055
Intervertebral disks	NR	0.176 ± 0.069	0.180	0.050	−0.050	0.312	0.633
Spinal cord	NR	0.135 ± 0.173	0.140	0.088	−0.010	−0.352	0.714
Subcutaneous fat	0.6 — 0.7^23^, ^41^	0.517 ± 0.155	0.550	0.215	−0.372	−0.111	0.171
Visceral fat	0.6 — 0.7^23^, ^41^	0.741 ± 0.200	0.700	0.344	−0.251	−0.714	0.452
Heart	NR	—	0	0	0	0	0
Muscle	0	0	0	0	0.292	−1.156	0.533
Internal Air	4.84^64^	—	4.84	4.84	0	0	0
External Air	9.2^35^	—	9.2	9.2	0	0	0

*Note*: (i) NR stands for non‐reported susceptibility values. (ii) The texture of 0‐fixed tissues (muscle and esophagus) was also modulated to avoid constant value regions. (iii) The experimental QSM value of muscle tissue was −0.226 ± 0.030 ppm. The References values column shows the Reported QSM values from the literature and the Experimental QSM values for HS, obtained from small ROIs as described in the Synthetic properties section.

Since the literature showed both, some variability in terms of the reported tissue susceptibilities and sometimes some inconsistencies with our experimental values (Table 1), we considered the following rules. If the experimental mean was consistent with the range reported in the literature, we used the computed value as the mean susceptibility of the tissue. If the experimental values were outside the literature range, the assigned mean susceptibility was a value between the experimental mean and the range limit, keeping it as close as possible to the latter. For the case of non‐reported tissues (e.g., spleen, stomach, and pancreas), we set the value of the mean susceptibility obtained from our experimental QSM, rounded to the second decimal place.

To avoid promoting over‐regularized solutions and to provide realistic textures for the synthetic QSM, we employed a similar strategy as that used in RC2.^35^ However, instead of using $R_{1}$ and $R_{2}^{*}$, the susceptibility of each voxel was modulated using $\rho_{\text{water}}$, $\rho_{\mathrm{fat}},$ and $R_{2}^{*}$. This heuristic was chosen based in the linear dependence found between $R_{2}^{*}$ and iron concentration,^12^, ^13^ and between susceptibility and PDFF^42^ Additionally, this formulation gives the practical advantage of separately modulate the textures related to water and fat structures in a same tissue class. To reduce scaling problems due to differences in the magnitude range of $R_{2}^{*}$, $\rho_{\text{water}}$, and $\rho_{\mathrm{fat}},$ we normalized each of these parameters in the 0 to 1 range, resulting in $r_{2}^{*}$, $\varrho_{W}$, and $\varrho_{F}$, respectively. We then defined the susceptibility with the following equation:

(1) $$\begin{array}{cc} \chi_{t}^{s}(r) & =\bar{\chi_{t}}+a_{t}\cdot \left(r_{2}^{*}(r)-\bar{r_{2,t}^{*}}\right)+b_{t}\cdot \left(\varrho_{W}(r)-\bar{\varrho_{W,t}}\right)+c_{t}\cdot \left(\varrho_{F}(r)-\bar{\varrho_{F,t}}\right) \end{array}$$

where $\chi_{t}^{s}\left(r\right)$ is the synthetic susceptibility of the tissue class t at the voxel r, $\bar{\chi_{t}}$ is the assigned mean susceptibility, and $\bar{r_{2,t}^{*}}$, $\bar{\varrho_{W,t}}$, and $\bar{\varrho_{F,t}}$ are the mean values taken from the whole mask of the tissue class t. The initial values of the texture modulation weighting parameters $a_{t}$, $b_{t}$, and $c_{t}$ were found by minimizing the normalized RMS error (nRMSE) between the synthetic susceptibility $\chi^{s}$ and the experimental QSM for every tissue class. Then, parameter values were manually adjusted, following a visual inspection heuristic, which consisted in increasing texture and structural details (e.g., small vessels and muscle fibers), commonly attenuated in the experimental QSM map due to over‐regularization. We modified the values of $a_{t}$, $b_{t}$, and $c_{t}$ to slightly increase the structural contrast, while avoiding the generation of piece‐wise constant tissue classes.

Sharp tissue transitions become unrealistic for an abdominal scenario. Tissue interphases are commonly smooth due to partial volume effects at the low acquisition resolutions employed for the abdomen. To reproduce these effects, we applied the approach described in RC2.^35^ A Gaussian kernel with σ = 0.5 was applied to each tissue class mask, except for hepatic veins, portal vein, large intestine, heart, and air regions. The probability $M_{t}\left(r\right)$ that a voxel r belongs to a tissue class t was calculated by dividing each resulting mask $M_{t}$ by the total sum of all tissue masks $M\left(r\right)=\sum_{t=1}^{23}M_{t}\left(r\right)$. Then,

(2) $$\chi^{s}\left(r\right)=\frac{1}{M\left(r\right)}\sum_{t=1}^{23}\chi_{t}^{s}\left(r\right)\cdot M_{t}\left(r\right).$$

#### 2.3.2 Synthetic $R_{2}^{*}$ map

For several pathologies, compromised tissues present significant changes not only in susceptibility values, but also in terms of signal decay,^43^ which results in signal void regions with poor SNR. We therefore defined a model to create a synthetic $R_{2}^{*}$ map based on the same expression of Eq. (1) but setting $b_{t}$ and $c_{t}$ to 0. Thus:

(3) $$\begin{array}{l} R_{2,t}^{*,s}\left(r\right)=\left\{\begin{array}{cc} R_{2}^{*}\left(r\right), & \text{if}\;m_{t}=0\\ \bar{R_{2,t}^{*}}+a_{t}\cdot \left(r_{2}^{*}\left(r\right)-\bar{r_{2,t}^{*}}\right), & \text{if}\;m_{t}=1 \end{array}\;\right. \end{array}$$

where $R_{2,t}^{*,s}\left(r\right)$ is the synthetic $R_{2}^{*}$ value of the tissue class t at the voxel r, and $\bar{R_{2,t}^{*}}$ is the mean $R_{2}^{*}$ value assigned to the tissue class t. The value of the $a_{t}$ parameter was chosen so that the resulting $R_{2,t}^{*,s}$ map acquired a realistic non‐smooth texture. When no $R_{2}^{*}$ changes are needed ($m_{t}=0$), the $R_{2}^{*}$ value is set as the experimental one. Otherwise, for each tissue class t the $R_{2}^{*}$ value is modified to an arbitrary value set by the user, to re‐create pathological scenarios.

### 2.4 Forward simulation

This is the final step of the phantom generation workflow, where the synthetic $\chi^{s}$ and $R_{2}^{*}$ maps are integrated into a signal model that also includes fat contributions. To eliminate the regions with fat swaps or lack of signal, we cropped five proximal and five distal axial slices of the complex water and fat images ($\rho_{W}$ and $\rho_{F}$) leaving a final matrix size of 192 × 192 × 80. The same cropping was applied to $\chi^{s}$ and $R_{2}^{*}$ .To simulate a complex acquired signal, we neglected $R_{1}$ and flip angle effects. Fat contributions were introduced into our signal model as a six‐peak model^38^ with frequency shifts ($f_{m}$) of −3.8, −3.4, −2.6, −1.94, −0.39, 0.6 ppm and relative amplitudes ($\alpha_{m}$) of 0.087, 0.693, 0.128, 0.004, 0.039, 0.048, respectively. The single‐ $R_{2}^{*}$ multipeak water‐fat signal model which describes the evolution of a voxel with time has been widely studied^19^, ^20^, ^22^, ^44^ and can be formulated as follows:

(4) $$\begin{array}{cc} S\left(r,TE_{k}\right) & =\left(\rho_{W}(r)+c_{k}\cdot \rho_{F}(r)\right)\cdot e^{\beta \cdot TE_{k}},\;\beta =i\cdot 2\pi \cdot f_{B}-R_{2}^{*,s}(r),\\ c_{k} & =\sum_{m=1}^{M}\alpha_{m}\cdot e^{i\cdot 2\pi \cdot B_{0}\cdot \gamma \cdot f_{m}\cdot TE_{k}},\;\text{with}\;\sum_{m=1}^{M}\alpha_{m}=1 \end{array}$$

where $S\left(r,TE_{k}\right)$ is the signal value of the voxel r at the k ‐th TE ($TE_{k}$), $B_{0}$ is the field strength, and γ is the gyromagnetic ratio. The fieldmap $f_{B}$ can be modeled as the 3D convolution of the local $\left(\chi_{\text{local}}^{s}\right)$ and background $\left(\chi_{\text{background}}^{s}\right)$ synthetic susceptibility maps with a magnetic dipole kernel D(r) along the *z*‐axis direction, which can be computed as a point‐wise multiplication in the Fourier domain.^45^, ^46^

(5) $$\begin{array}{ll} & f_{B}=\gamma \cdot B_{0}\cdot {ℱ}^{-1}\;\left[ℱ\left(\chi_{\text{local}}^{s}\left(r\right)+\chi_{\text{background}}^{s}\left(r\right)\right)\cdot ℱ\left(D\left(r\right)\right)\right] \end{array}$$

with ℱ and $F^{-1}$ the direct and inverse Fourier transform, respectively.

Instead of using the classical continuous formulation,^45^, ^46^ we employed a discrete approach^47^ and zero‐padding by a factor of 2 along each dimension. This alternative formulation reduces aliasing artifacts that appear around regions with abrupt susceptibility variations (e.g., fat, bone, and air interfaces), which are originated by the sampling processes.^48^

### 2.5 Phantom generation

#### 2.5.1 Synthetic maps scenarios

To verify the ability of our phantom to simulate different susceptibility and $R_{2}^{*}$ values, we defined three scenarios:

- **Healthy subject (HS):** $\bar{\chi_{t}}$ values were assigned following the procedure described in the Synthetic susceptibility map section. In this case, the $R_{2}^{*}$ map was kept as the experimental one for every tissue class.
- **Pathologic lobe (PL):** This scenario aims at showing the behavior of the phantom when all susceptibility values were slightly modified, while $R_{2}^{*}$ values increase significantly. In this case, each $\bar{\chi_{t}}$ value was assigned as the mean value of the experimental QSM, instead of spherical ROIs described in the Synthetic susceptibility map section. These alternative values were taken from the whole mask of the tissue class t, similar as $\bar{r_{2,t}^{*}}$, $\bar{\varrho_{W,t}}$, and $\bar{\varrho_{F,t}}$ in Eq. (1). The values of susceptibility model parameters $a_{t}$, $b_{t}$, $c_{t}$ were the same as in HS. We simulated a lobe with a mean $R_{2}^{*}$ value of 150 $s^{-1}$ and a mean susceptibility value of 0.2 ppm located in the liver, close to the porta hepatis. For the rest of the tissues, the $R_{2}^{*}$ values were the same as in HS.
- **Iron overload (IO):** This phantom simulates a pathologic scenario, where $\bar{\chi_{t}}$ and $\bar{R_{2,t}^{*}}$ values were drastically increased for the affected tissues. Liver, spleen, pancreas, and vertebrae were chosen as the compromised tissues, as they all can be observed in a single slice. This represents a realistic scenario for hemochromatosis or hemosiderosis, where iron overload occurs in the liver, spleen, pancreas, and bone marrow, causing higher susceptibility values and a faster $R_{2}^{*}$ decay.^43^ In this case, the susceptibility model parameters $a_{t}$, $b_{t}$, and $c_{t}$ were set manually, attempting to reproduce realistic textures. For non‐affected tissues, the values of those parameters were the same as in HS.

Table 1 shows the reference values and the model parameters employed in the generation of HS and PL. Table 2 shows the susceptibility and $R_{2}^{*}$ model parameters employed in IO.

**TABLE 2:** Synthetic susceptibility and $R_{2}^{*}$ model parameters employed for the IO phantom

	Synthetic susceptibility model				Synthetic $R_{2}^{*}$ model	
Tissue	$\bar{\chi_{t}}$(ppm)	$a_{t}$(ppm)	$b_{t}$(ppm)	$c_{t}$(ppm)	$\bar{R_{2}^{*}}$(Hz)	$a_{t}$(Hz)
Liver	0.60	−0.332	0.719	0.854	100	225
Spleen	0.28	0.226	0.076	0.653	54	100
Pancreas	0.20	0.528	−1.523	−0.015	57	110
Vertebrae	0.05	0.111	−0.251	−1.055	145	200

To simulate the background field effects, we considered the external air, the large intestine and the gases from the gastrointestinal tract as background susceptibility sources. In order to reproduce a realistic background scenario, we appended axial‐flipped copies of our phantom in the axial direction, to emulate the superior and inferior regions of the body outside the phantom ROI. We also zero‐padded along the sagittal and coronal directions, resulting in a matrix size of 576 × 576 × 240. Then, we performed the dipole convolution and cropped the resulting field $f_{B}$ to its original size.

#### 2.5.2 Simulated acquisitions

We simulated two acquisition protocols, which correspond to two of the most common approaches employed for abdominal QSM: water/fat separation and in‐phase acquisitions. The former consists in acquiring a series of echoes at a short and regular TE spacing and estimate the fieldmap using iterative algorithms like IGC. The latter approach sets the acquisitions at the TEs when water and fat are in the same phase. These TEs are commonly defined using a single‐peak fat model assumption, which results in echo acquisitions at every 2.3 ms for a 3T scanner. Given the signal model of Eq. (4), the simulation of these protocols is straight‐forward, and it just needs to set the TEs, defined as follows:

- **Protocol 1 (P1):** Six‐echo acquisition with the same TEs as the experimental data: TE_1_ = 1.8 ms and Δ TE = 1.56 ms. With this protocol we performed a QSM pipeline with a chemical shift correction step and compared the simulated phantom appearance with the experimental data.
- **Protocol 2 (P2):** Five in‐phase echoes with TE_1_ = 2.3 ms and ΔTE = 2.3 ms. With this protocol we performed a direct QSM reconstruction approach, without need of any chemical‐shift correction.

### 2.6 Experimental validation

For this implementation, we assumed a perfect background‐field removal. Thus, all voxels corresponding to air regions and large intestine were set to 0 and masked out from the synthetic maps and the signal model ($\chi_{\text{background}}^{s}=0$). The synthetic susceptibility phantom was demeaned and forward‐simulated as described in Eq. (4). We decided to keep the phase contributions of fat, to study how the errors from the water/fat separation and in‐phase approaches propagate and affect the QSM reconstructions.

We performed the dipole inversion with five different methods reported in literature: Morphological Enabled Dipole Inversion (MEDI),^49^ STreaking Artifact Reduction (STAR‐QSM),^50^, ^51^ Nonlinear Dipole Inversion (NDI),^52^ FANSI,^34^, ^53^ and Hybrid Data fidelity term approach for QSM (HD‐QSM).^54^ To evaluate our phantom as a ground truth for the assessment of QSM algorithms, we compared the quality of the obtained reconstructions under two different schemes:

- **QSM with chemical shift correction:** Using data from P1, we performed a chemical shift correction and a water/fat separation with IGC^22^ and T2*‐IDEAL.^39^ The resulting $B_{0}$ fieldmap was employed as input for the dipole inversion. For NDI, FANSI, and HD‐QSM we defined a magnitude‐based and spatially variable weight for the data fidelity as:

(6) $$\begin{array}{ll} & W=\frac{\sum_{i=1}^{N}{\left|S\left(TE_{i}\right)\right|}^{2}\cdot TE_{i}}{\sum_{i=1}^{N}\left|S\left(TE_{i}\right)\right|\cdot TE_{i}} \end{array}$$

where *N* is the number of echoes.

- **Direct QSM:** Using data from P2, we performed a standard QSM pipeline. This includes phase unwrapping with ROMEO,^55^ a linear least squared multi‐echo fit, and then dipole inversion. For the data fidelity term (NDI, FANSI, and HD‐QSM), we considered the spatially variable weight W, defined in Eq. (6).

We added normally distributed complex noise (σ=1) to the simulated data, with a peak SNR of 100. Reconstructed fieldmaps, $R_{2}^{*}$ maps and QSM maps were compared against ground truths using the nRMSE metric. To compare QSM reconstructions, we also employed the QSM‐specific structural similarity metric (XSIM).^56^ To obtain the optimal QSM reconstructions (NDI, MEDI, FANSI, and HD‐QSM), parameters were set as those that minimize nRMSE.

## 3 RESULTS

### 3.1 Phantom simulations

**FIGURE 2:**
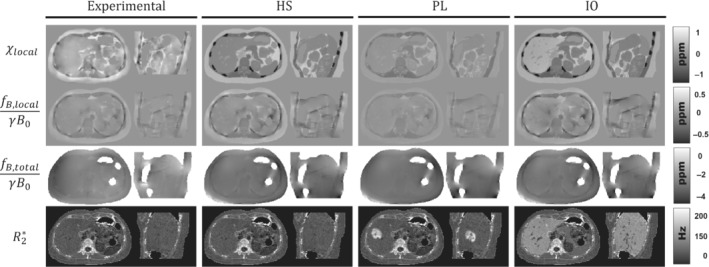
Representative axial and sagittal slices of the susceptibility, local field, total field, and $R_{2}^{*}$ maps of the experimental data and the three simulated phantoms: HS, PL, and IO

Figure 2 shows a comparison between experimental data and the three synthetic phantoms. The first row shows an axial slice of the experimental susceptibility and the synthetic susceptibilities for phantoms HS, PL, and IO. HS shows a high contrast between the different tissue classes and texture details in tissues like pancreas, muscle, kidney, and visceral fat. In PL, instead of using specific ROIs, the mean susceptibilities were assigned as the average values inside each tissue class. This resulted in lower contrast between the tissue classes, especially in the bone/muscle, muscle/fat, and organ/fat transitions. The IO phantom aimed to simulate an iron overload scenario, with iron accumulations in the liver, spleen, pancreas, and vertebrae, as can be seen in the top‐right of Figure 2. Here, the liver shows a significant susceptibility increase, whereas smaller increases occur for the rest of the compromised tissues.

The second and third rows of Figure 2 show axial and sagittal slices of the resulting local and total fieldmaps after performing the convolution with the dipole kernel. For local fieldmaps, HS and IO present a similar level of detail as that in the experimental case, with higher contrast in the muscle/bone interphase and in the hepatic veins of IO. In the case of PL, the assigned $\bar{\chi_{t}}$ values of muscle, ribs, and fat are closer to each other (Table 1), which results in a smoother transition between these tissue classes with no hyperintense regions around the ribs, in contrast to experimental data, HS, and IO. For total fieldmaps, the three phantoms show a similar appearance compared to the experimental case, except for the two hyperintense regions around the masked intestines and at the posterior‐left side of the phantoms.

The last row of Figure 2 shows the synthetic $R_{2}^{*}$ maps. For HS, $R_{2}^{*}$ map is the same as in the experimental case. For PL and IO, the maps show increased $R_{2}^{*}$ values for the simulated lobe and the iron overload in the chosen tissues, while keeping similar textures compared with the experimental case.

**FIGURE 3:**
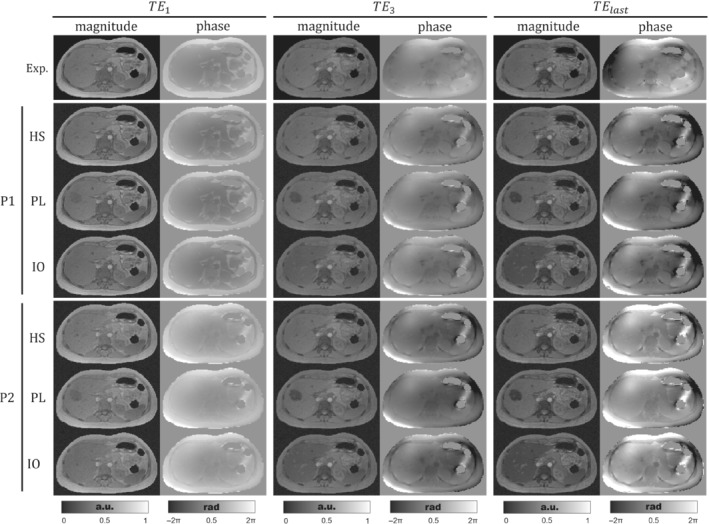
Axial views of the experimental and simulated acquired signals for phantoms HS, PL, and IO with protocols P1 and P2. The three pairs of columns show magnitude and the unwrapped phase at the first, third, and last TE (sixth echo for P1 or fifth for P2), respectively. To ease visualization, magnitude images were normalized between 0 and 1.

Figure 3 shows the resulting magnitudes and the unwrapped phases using ROMEO^55^ for the three phantoms, after performing the forward simulation with the acquisition protocols P1 and P2. On the one hand, for all phantoms the phase of P1 acquisitions shows a similar appearance as that of the experimental case at the first echoes, with the same exceptions mentioned for Figure 2. For higher TEs, phase images become different compared to the experimental case, with hyperintense regions surrounding the subcutaneous fat. These differences might be explained by the simplifications made for modeling the background susceptibility sources. On the other hand, P1 magnitude images are almost identical to those of the experimental case, particularly the same out‐of‐phase artifacts at the muscle/fat interphases and at the right‐hand side of the pancreas at $TE_{1}$. For PL and IO images, a faster signal decay is observed in the simulated lobe and the tissues with iron overload: liver, spleen, pancreas, and vertebrae.

Protocol P2 was defined to simulate in‐phase acquisitions. In the first TE, the above‐mentioned out‐of‐phase artifacts are significantly reduced for P2 magnitude images. Additionally, the P2 phase image at $TE_{1}$ shows a reduced water/fat contrast compared with P1 and the experimental data. For experimental data and P1, the third and last TEs are close to in‐phase acquisition times ($TE_{3}$ = 4.92 ms and $TE_{\text{last}}$ = 9.6 ms). As consequence, experimental data, HS‐P1, and HS‐P2 show similar magnitude and phase images for $TE_{3}$ and $TE_{\text{last}}$. The last echo phase image of P2 starts showing a higher contrast between fat and water structures; this can be clearly seen in the fat/muscle interphase.

### 3.2 Phantom reconstructions

**FIGURE 4:**
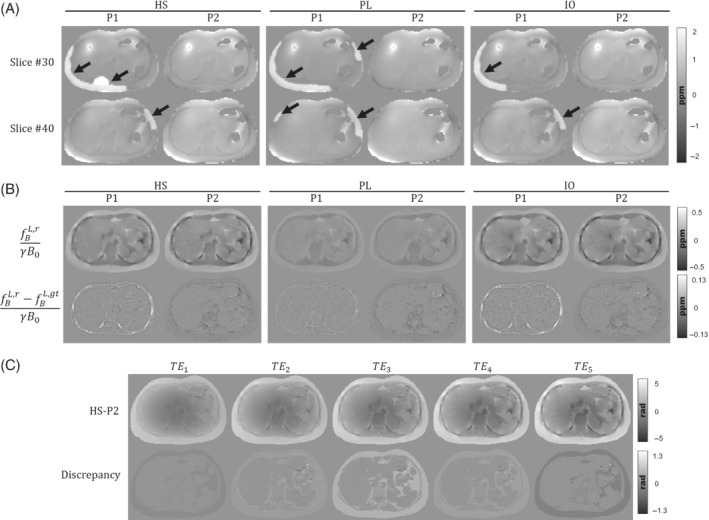
(A) Two axial examples of the total fieldmap estimations of phantoms HS, PL, and IO, for the corresponding acquisition protocols P1 and P2. Estimation errors from IGC can be seen as hyperintense regions around the subcutaneous fat (black arrows). (B) Axial slices of the local field reconstructions for phantoms HS, PL, and IO, and their difference with ground truth. Left and right columns show the results for P1 and P2 protocols, respectively. (C) Unwrapped phase of the HS phantom with the one‐peak‐based in‐phase protocol (P2) and its discrepancies with a perfect in‐phase simulation at every TE

Figure 4(A) shows the estimated total fieldmaps (HS, PL, and IO) for the two strategies associated to P1 and P2. For the in‐phase protocol, total fieldmap estimations using ROMEO and multiecho fitting results in smooth and continuous fieldmaps without phase wrap errors. However, IGC estimations for P1 show several fieldmap errors in some regions (pointed by black arrows), showing the sensitivity of the algorithm under the presence of strong fieldmap inhomogeneities. Given the significative differences between P1 and P2 total field estimations, we decided to ignore this step for the subsequent QSM reconstructions, and assume a perfect background field removal scenario, as described in the Experimental validation section.

Figure 4(B) shows the reconstructed local fieldmaps for both protocols and their respective differences against ground truths. For the three phantoms, reconstructions with protocol P1 produce fieldmaps with overestimated values in the ribs. This might be caused by residual errors from IGC and $T_{2}^{*}$‐IDEAL that might propagate to the dipole inversion step. Reconstructions with protocol P2 show structural differences in the fatty tissue regions (i.e., subcutaneous fat, visceral fat, and bone marrow in vertebrae). We compared the one‐peak based acquisitions of HS‐P2 against a perfect in‐phase simulation where we forced $c_{k}=1$ for each k in Eq. (4). The difference maps of Figure 4(C) show the discrepancies generated under the one‐peak based in‐phase assumption, where the main differences can be appreciated in the fat‐related tissues.

**FIGURE 5:**
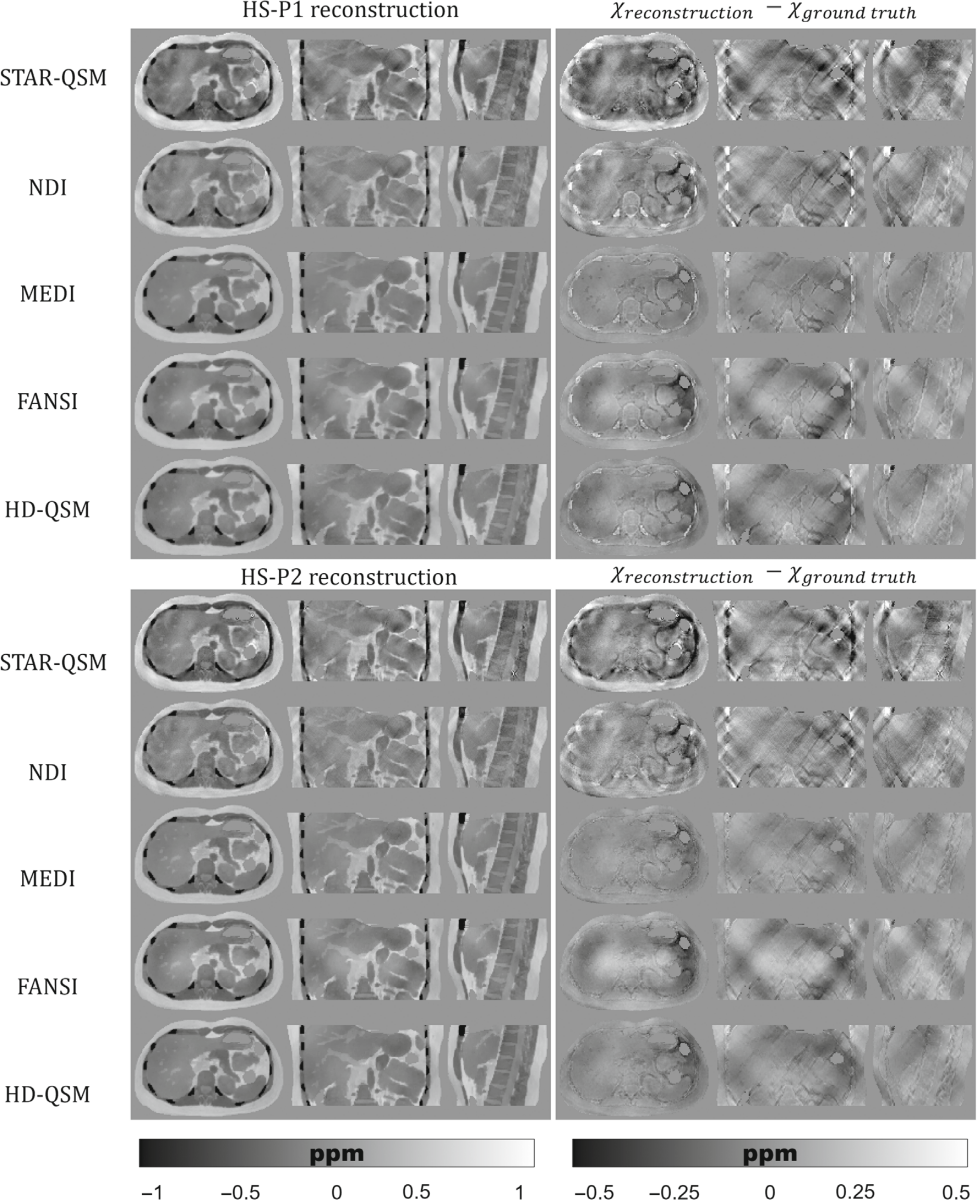
(Left) Axial, coronal, and sagittal slices of QSM reconstructions for the HS phantom with P1 (top) and P2 (bottom). (Right) Axial, coronal, and sagittal slices with the difference between the reconstructed QSM image and the ground truth

**FIGURE 6:**
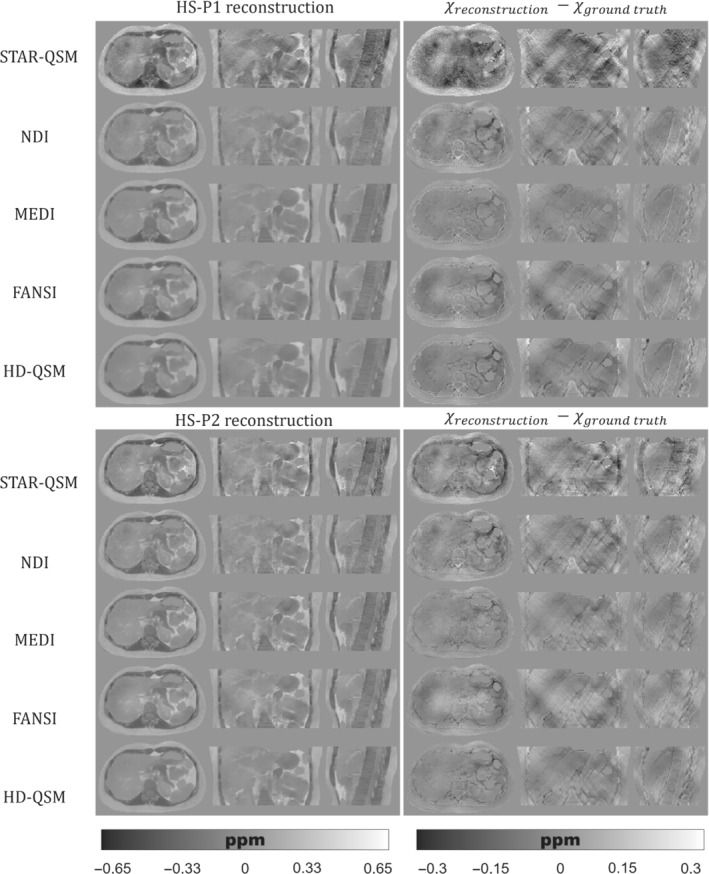
(Left) Axial, coronal, and sagittal slices of QSM reconstructions for the PL phantom with P1 (top) and P2 (bottom). (Right) Axial, coronal, and sagittal slices with the difference between the reconstructed QSM image and the ground truth

**FIGURE 7:**
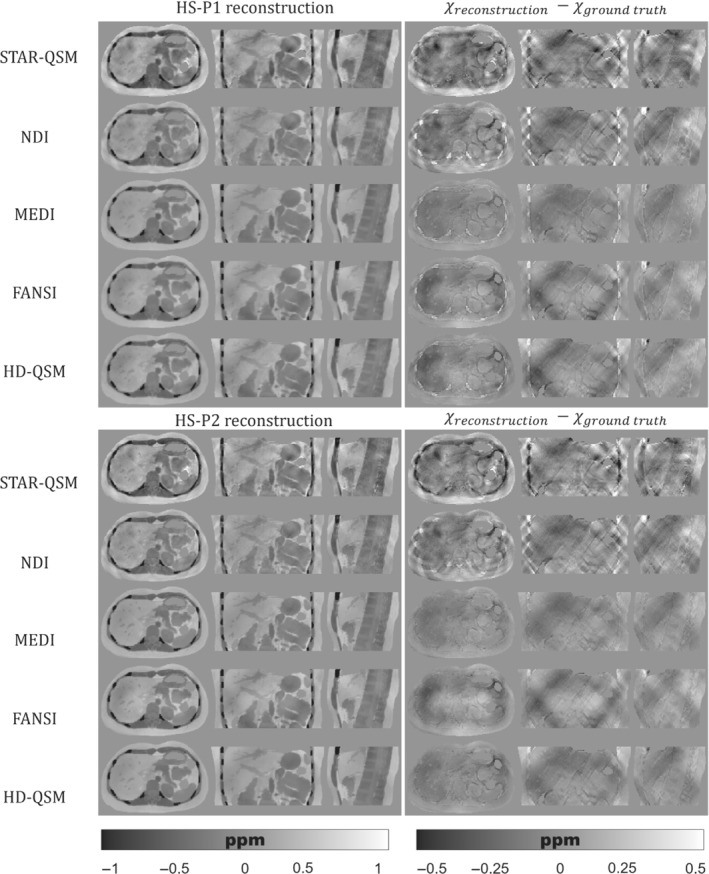
(Left) Axial, coronal, and sagittal slices of QSM reconstructions for the IO phantom with P1 (top) and P2 (bottom). (Right) Axial, coronal, and sagittal slices with the difference between the reconstructed QSM image and the ground truth

Figures 5, 6, 7 show the QSM reconstructions of the HS, PL, and IO phantoms. For HS, PL, and IO, the STAR‐QSM and NDI reconstructions present several streaking artifacts around ribs and vertebrae. With MEDI, FANSI, and HD‐QSM, reconstructions show a significant reduction of streaking artifacts, but also some dark regions around the ribs and a loss of structural information in the regions surrounding the stomach, intestine, left kidney, ribs, and vertebrae. Whereas STAR‐QSM present larger susceptibility values for the ribs and subcutaneous fat compared to the ground truth, the other reconstruction algorithms present smaller susceptibility values, resulting in hyperintense structures in the difference images, especially with NDI. For the above‐mentioned regions, P1 reconstructions present larger structural errors than P2. This might be a consequence of error propagation from the previous fieldmap estimation step with IGC and $T_{2}^{*}$‐IDEAL. Nevertheless, it is interesting how STAR‐QSM manages to address that problem in P1 but generates the opposite problem in P2, with hypointense regions around the whole rib cage.

Table 3 shows the nRMSE of the reconstructed fieldmaps and $R_{2}^{*}$ maps. It also shows the nRMSE and XSIM of the QSM reconstructions for each evaluated method. For $R_{2}^{*}$ maps and fieldmaps, reconstructions present a similar nRMSE, with slightly higher values for the P2 protocol. In the case of the fieldmap, PL‐P2 presents a significantly higher nRMSE. This might be explained by the presence of unreliable voxels from the phase unwrapping of echo fitting steps, which increase severely the nRMSE but do not affect significantly the respective QSM reconstructions, compared with PL‐P1. Despite having a similar appearance compared with HS and IO reconstructions, PL shows a significant nRMSE increase. This might be explained because of the shorter range of susceptibility values in PL, which decreases the contrast between tissues and increases the effects of noise.

**TABLE 3:** Accuracy metrics for the reconstructed fieldmaps, $R_{2}^{*}$ and QSM

		HS		PL		IO	
		P1	P2	P1	P2	P1	P2
Fieldmap	nRMSE (%)	14.50	19.38	18.33	40.64	14.50	18.57
$R_{2}^{*}$	nRMSE (%)	13.48	14.47	13.16	14.21	12.12	12.98
STAR‐QSM	nRMSE (%)	51.18	52.64	82.28	71.93	54.41	55.87
	XSIM	0.472	0.468	0.340	0.423	0.437	0.444
NDI	nRMSE (%)	41.80	39.84	47.38	48.85	46.10	43.50
	XSIM	0.519	0.522	0.461	0.470	0.468	0.476
MEDI	nRMSE (%)	25.41	22.84	36.29	35.84	30.29	27.58
	XSIM	0.721	0.734	0.574	0.621	0.662	0.678
FANSI	nRMSE (%)	29.92	25.88	43.32	41.59	33.35	29.43
	XSIM	0.659	0.704	0.525	0.561	0.611	0.655
HD‐QSM	nRMSE (%)	32.56	28.94	50.42	43.29	36.64	33.08
	XSIM	0.654	0.702	0.457	0.542	0.597	0.644

As qualitatively observed in Figure 5 and Figure 7, the TV regularization‐based methods (MEDI, FANSI, and HD‐QSM) show improved reconstructions compared with STAR‐QSM and NDI, with similar nRMSE and XSIM values around 25%–36% and 0.60–0.75, respectively.

## 4 DISCUSSION

In this work, we presented a realistic numerical phantom of the abdomen for the evaluation and comparison of different QSM reconstruction methods and pipelines under controlled conditions. The phantom effectively simulates susceptibility and $R_{2}^{*}$ values, and their corresponding magnitude and phase signals considering fat contributions and background susceptibility sources. It shows a realistic texture appearance and out‐of‐phase effects. The publicly available data and source codes were developed in a modular and flexible pipeline, where the different features can be easily included or removed (e.g., background susceptibility sources). This allows users to simulate phantoms with different tissue properties, signal effects, and acquisition TEs.

Our simulation pipeline employs MRI‐based segmentations and a multiparametric model to provide realistic geometries and textures to each of the tissue classes. These features represent an advantage compared with simplified models based in simple geometric shapes, or the current abdominal models with realistic anatomies but piecewise tissue property values.^19^, ^36^, ^57^ Additionally, the fact that synthetic $R_{2}^{*}$ maps can be easily adjusted allows the users to simulate realistic scenarios, where not only the susceptibility but also the signal decay is involved. Both $R_{2}^{*}$ and QSM values can be easily and arbitrary modified, beyond the default parameters described here. Thus, users can modify or correct these rules and values according to their needs.

The addition of fat signal effects represents a new approach for the evaluation of the current outside‐the‐brain QSM pipelines. On the one hand, this feature allows researchers to develop new algorithms for simultaneous QSM in the presence of fat, and to compare different reconstruction pipelines which in some cases require different acquisition times to remove the chemical shift effects (e.g., $T_{2}^{*}$‐IDEAL, in‐phase acquisitions, multispectral ARMA modeling,^58^ etc.). Our model allows us to define arbitrarily the TEs of the simulated acquisitions. In practice, this can be done simply by specifying the TEs in the header of our source code. On the other hand, this feature, in combination with the simulation of different *χ* and $R_{2}^{*}$ values, could be used to generate training data for neural‐network‐based aproaches, as proposed by Zhao et al.^36^ Additionally, the phantom also includes a fieldmap and a PDFF ground truths, to evaluate, identify, and solve critical stages of the employed pipelines and water‐fat separation methods.

We used a forward simulation scheme to evaluate two of the current outside‐the‐brain QSM pipelines: water‐fat separation and in‐phase acquisition. For water‐fat separation with IGC and $T_{2}^{*}$‐IDEAL, phantom reconstructions showed that the algorithm is significatively sensitive to strong fieldmap inhomogeneities, and it is also prone to leave residual errors in regions with drastic susceptibility changes, like ribs and muscle/fat interphases. These errors were propagated to the dipole inversion step and generated systematic structural differences between the reconstructions and the ground truth for most QSM dipole inversion algorithms.

For in‐phase acquisitions, the reconstructed fieldmaps presented structural errors around the fat regions. These errors might be explained by TE mismatch between the one‐peak‐based in‐phase acquisition and the multiple frequency shifts of the six‐peak fat model, causing fat discrepancies as shown in Figure 4(C). Despite being the main clinical workhorse for water and fat images, questions have been raised about the usage of one‐peak‐based acquisitions and how this scheme introduces significant quantifications bias for QSM.^19^ In this sense, the effective TEs proposed by Boehm et al.^19^ could be employed in a future work, in order to evaluate a potential reduction on these mismatch effects.

QSM reconstruction experiments showed that algorithms like MEDI, FANSI, and HD‐QSM were more robust to noisy voxels and streaking artifacts. However, current algorithms are still prone to errors in regions with drastic susceptibility changes. This could be seen in Figures 5, 6, 7, where structural errors in the ribs were present in both acquisition protocols P1 and P2, despite of being significantly reduced for P2. These results show the potential of our phantom as a ground truth, allowing researchers to identify and solve critical stages of the current QSM pipelines.

There are some limitations in our framework. Due to the absence of an abdominal MRI atlas for the registration of the different tissue classes, the segmentations were performed just for common abdominal structures of a single female individual. Considering anatomical variations would require repeating the entire segmentation process.

Our signal model of Eq. (4) is based in the standard formulation employed in current water/fat separation state‐of‐the‐art studies,^19^, ^20^, ^22^, ^44^ which neglect $R_{1}$ and flip angle effects. Including these effects would be desirable to improve simulations and to allow and compare new acquisition scenarios. However, the incorporation of the previous effects is not straightforward (particularly $R_{1}$) since one would need to generate the corresponding map using the same acquisition as that used for $R_{2}^{*}$ and QSM, and thus, ensuring spatial correspondence among all three parameters may be difficult to achieve. This might be realized by using fingerprinting acquisitions,^59^, ^60^ which enables fast multiparametric maps (compatible with a breath hold scan) or using self‐navigating sequences^61^ that could allow free‐breathing multiparametric acquisitions.

Our simulation corresponds to a static breath‐hold case, without moving artifacts from small respiratory motion. Additionally, the water and fat fractions are also static, leading to just one PDFF case. If necessary, incorporating the respiratory motion might be addressed using a similar model as that proposed by Lo et al.^57^ Considering dynamic PDFF might be solved by employing a synthetic PDFF and including it in the signal model formulation of Eq. (4) and its contributions to the synthetic $R_{2}^{*}$ map.^62^ The modular framework of our phantom source code allows including these features, and this might be considered as a future work.

## 5 CONCLUSIONS

We developed a numerical abdominal phantom for QSM, which effectively simulates susceptibility and $R_{2}^{*}$ maps with realistic texture appearance. Our simulation pipeline included two adjustable tissue properties and a signal model that incorporates background fields and fat phase contributions, allowing to recreate different susceptibility and signal scenarios and to mimic some abnormal conditions. Our reconstruction experiments show the potential of our phantom as a ground truth to develop and compare not only QSM algorithms but also water‐fat separation methods.

## Data Availability

The source code is publicly available at: https://gitlab.com/qsm1/realistic_abdominal_phantom.
